# Case report: Drug-eluting bead transcatheter arterial chemoembolization in liver metastasis and retroperitoneal lymph node metastases of renal cell carcinoma: effective local therapy with the first report

**DOI:** 10.3389/fonc.2024.1371414

**Published:** 2024-06-21

**Authors:** Zhenkang Qiu, Junjie Ji, Shuo Zhang, Song Wang

**Affiliations:** ^1^ Interventional Medical Center, The Affiliated Hospital of Qingdao University, Qingdao, Shandong, China; ^2^ Department of Urology, The Affiliated Hospital of Qingdao University, Qingdao, Shandong, China

**Keywords:** DEB-TACE, renal cell carcinoma, livermetastasis, retroperitoneal lymph node metastases, case report

## Abstract

The liver is the fourth most common site of metastasis in renal cell carcinoma (RCC), which is usually treated with systemic therapies and local treatments. However, local treatments are challenging in RCC patients with liver metastasis who failed in first-line systemic therapy. Here, we report a case of a patient with both liver-dominant RCC metastasis and recurrence in the operative site who had failed in first-line targeted therapy plus immunotherapy, received drug-eluting bead transcatheter arterial chemoembolization (DEB-TACE), and achieved a complete response.

## Case report

Drug-eluting bead transcatheter arterial chemoembolization (DEB-TACE) is a technique that allows drugs to be released slowly into the tumor tissue after artery embolization, which differs from traditional transcatheter arterial chemoembolization (TACE) (1). The liver is the fourth most common site of metastasis in renal cell carcinoma (RCC) (2), which is usually treated with systemic therapies and local treatments. However, local treatments are challenging in RCC patients with liver metastasis who failed in first-line systemic therapy. Here, we report a case of a patient with both liver-dominant RCC metastasis and recurrence in the operative site who had failed in first-line targeted therapy plus immunotherapy, received DEB-TACE, and achieved a complete response.

**Figure 1:**
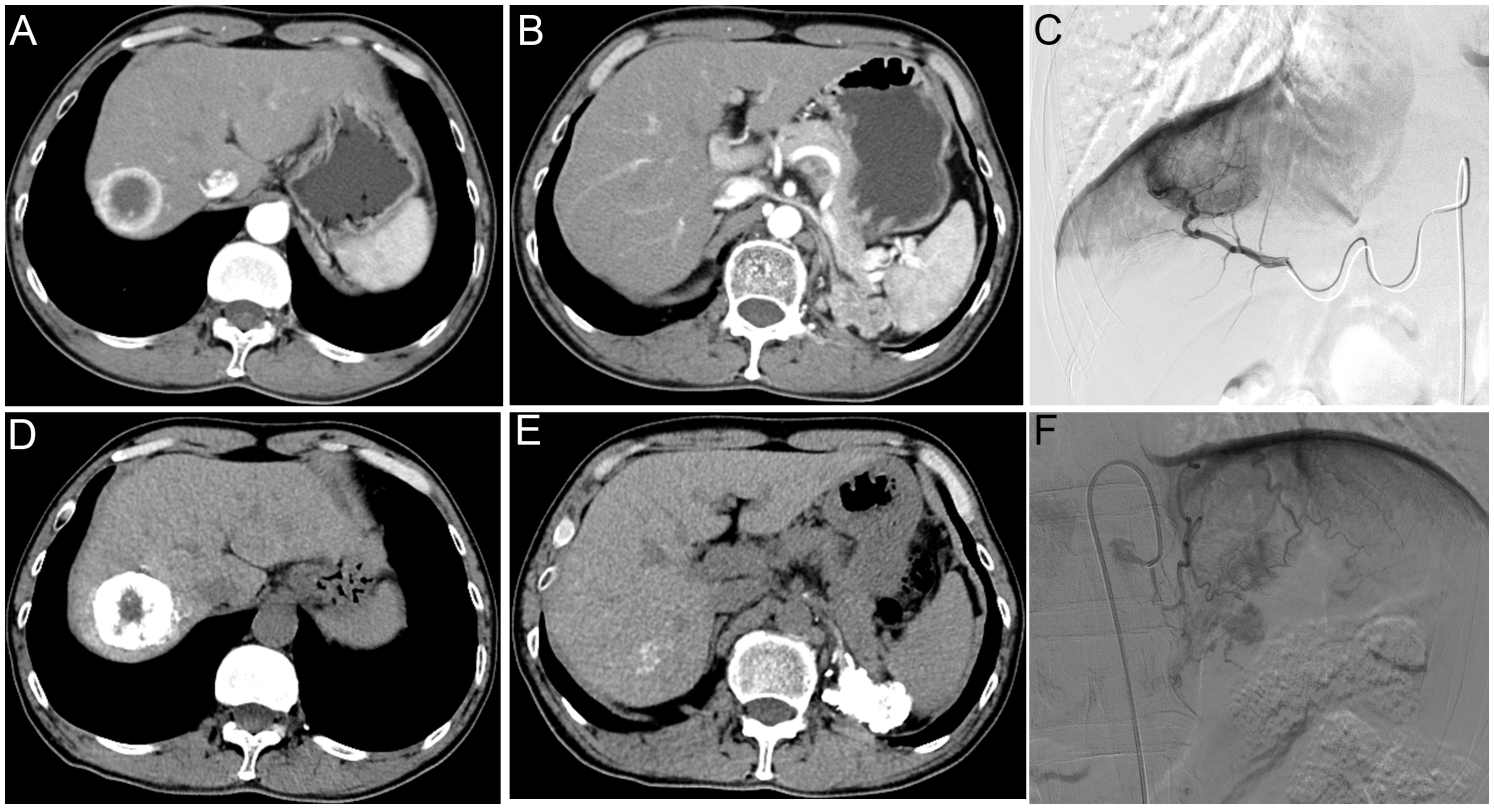
A 52-year-old man with liver metastasis and retroperitoneal lymph node metastases of renal cell carcinoma received drug-eluting bead transcatheter arterial chemoembolization. Preoperative CT enhanced scanning images of arterial phase **(A, B)**. Intraoperative angiography showed apparent tumor staining **(C, F)** and lipiodol deposition after embolization **(D, E)**.

A 52-year-old man was diagnosed with renal cell carcinoma (RCC) and underwent a left radical nephrectomy. The pathology showed clear cell RCC with the pT1N0M0 stage. He developed liver metastasis 16 months after the surgery. Therefore, he received sunitinib plus tislelizumab as first-line systemic therapy. Due to the severe adverse reactions of thrombocytopenia, the treatment was changed to axitinib plus tislelizumab. Six months later, he underwent enhanced CT as a routine examination. The CT revealed multiple highly enhanced masses in the left renal region resembling enlarged retroperitoneal lymph nodes (**Figure 1A**) and a ring-enhanced mass with a 3.75 cm maximum diameter in segments VII and VIII of the liver (**Figure 1B**). He was finally diagnosed with liver and retroperitoneal lymph node metastases, which indicated the failure of first-line targeted therapy plus immunotherapy.

The digital subtraction angiography indicated that the liver metastasis and retroperitoneal lymph node metastases were supplied by the right branch of the proper hepatic artery (**Figure 1C**) and the left inferior phrenic artery (**Figure 1F**), respectively. Then, he underwent drug-eluting bead transcatheter arterial chemoembolization (DEB-TACE) with two 50 mg doxorubicin-loaded callispheres of low diameter (70–150 μm) and TACE using a mixture of 2 mg raltitrexed and 7 ml lipiodol in each targeted artery (**Figures 1D, E**).

**Figure 2:**
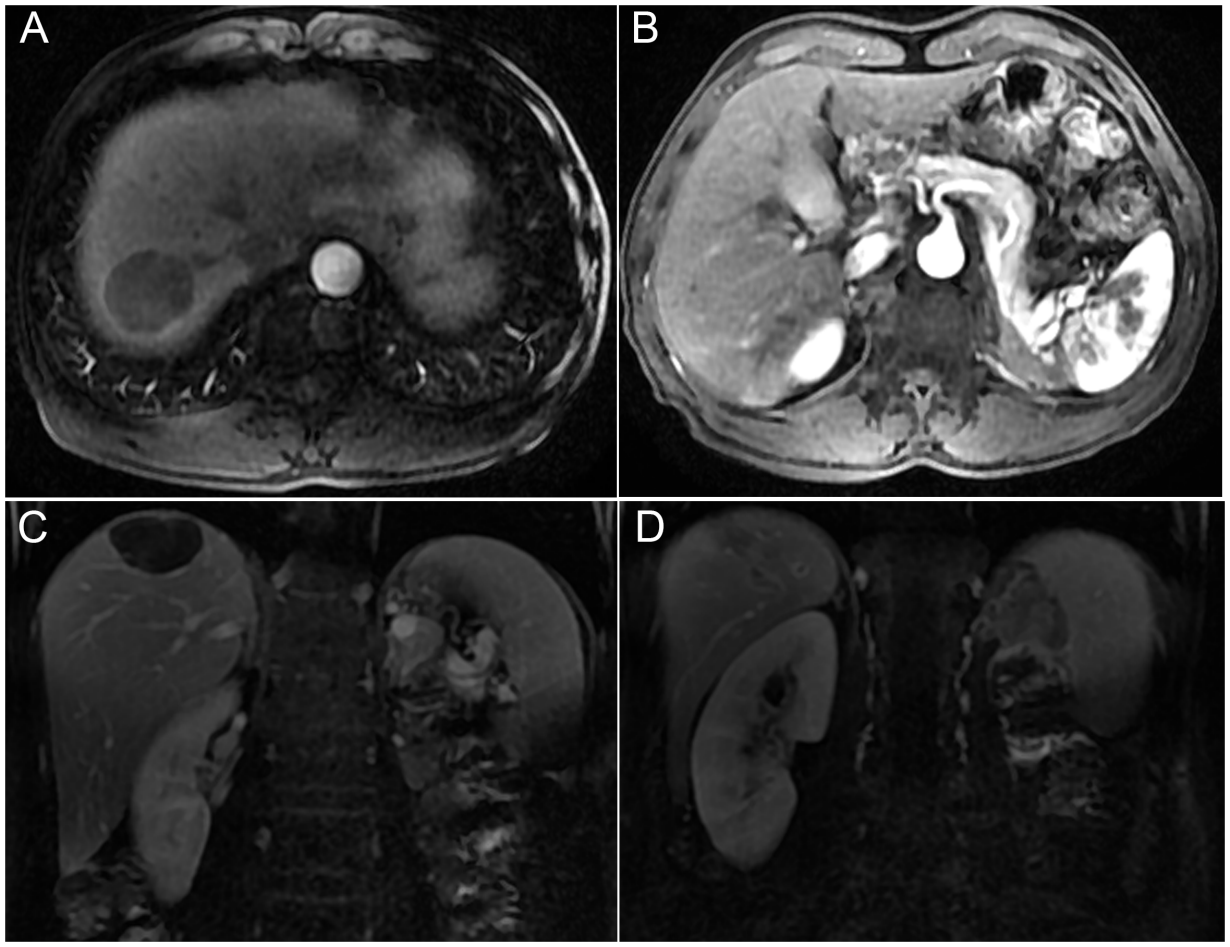
The enhanced MR images showed a complete response of the tumor after six months of follow-up. Axial and coronal MR images showed no tumor activity in both liver metastasis **(A, C)** and left retroperitoneal lymph node metastases **(B, D)**.

**Figure 3:**
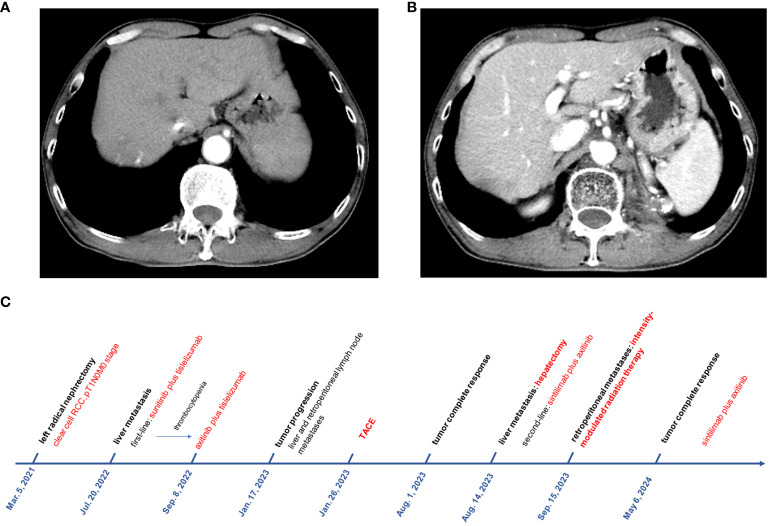
The enhanced CT scan showed no tumor activity in both liver metastasis **(A)** and left retroperitoneal lymph node metastases **(B)** after 15 months of follow-up. **(C)** The timeline with relevant events.

The patient had mild upper abdominal pain symptoms after surgery, which disappeared on the third day after surgery. After six months of follow-up, the enhanced MR scan showed no tumor activity in both liver metastasis (**Figures 2A, C**) and left retroperitoneal lymph node metastases (**Figures 2B, D**), indicating the complete response of this patient treated with DEB-TACE according to the Modified response evaluation criteria in solid tumors. After a multi-disciplinary discussion, the patient received a partial hepatectomy of segment VII. Postoperative pathology showed infiltration of poorly differentiated cancer with extensive necrosis in the liver tumor tissue, which was classified as metastatic clear cell carcinoma (WHO/ISUP nuclear grading: grade 3). The retroperitoneal lymph node metastasis area received intensity-modulated radiation therapy of 6MV X-ray DT (High-risk area: 5500cGy/220cGy/25f/5w; Low-risk area: 5150cGy/206cGy/25f/5w). The system treatment was replaced with sintilimab 200mg q3w plus axitinib 5mg bid for 28 days. After 15 months of follow-up, the enhanced CT scan showed no tumor activity in both liver metastasis (**Figure 3A**) and left retroperitoneal lymph node metastases (**Figure 3B**). The timeline with relevant events is shown in **Figure 3C**. The patient is very satisfied with the treatment effect and believes that this minimally invasive intervention treatment should be performed in suitable metastatic RCC patients, bringing benefits to more patients.

TACE is a traditional therapy for hepatocellular carcinoma, with advantages in high local efficacy, small wound, and early recovery (3). For RCC patients with local recurrence or liver metastasis after radical nephrectomy, systemic targeted therapy, immunotherapy, and their combination are first-line recommendations in the 2022 European Association of Urology Guidelines (4). However, local treatment is not recommended in metastatic RCC patients with failed first-line systemic treatment, and DEB-TACE has not been previously reported for use in local recurrent metastases of RCC patients after first-line systemic therapy. Our patient received DEB-TACE in both liver metastasis and retroperitoneal lymph node metastases after failing targeted therapy plus immunotherapy, and surprisingly achieved a complete response. The reason might be the highly hypervascular nature of RCC metastases that enables the embolization of targeted arteries to block the whole blood flow of tumor tissue (5). On the other hand, the slow and continuous drug release of DEB-TACE could bring better efficacy than traditional TACE. Although DEB-TACE showed an excellent curative effect, continuous systemic therapy, and regular reexamination should not be neglected. Thus, it is very important to conduct multi-disciplinary treatment at the appropriate time. This patient benefits from DEB-TACE, surgical resection, radiation therapy, and systematic treatment.

DEB-TACE showed excellent efficacy in an RCC patient with liver metastasis and retroperitoneal lymph node metastases after failing first-line targeted therapy plus immunotherapy, which was not previously reported and could be recommended in metastatic RCC with failed systemic therapy. More high-quality randomized controlled trials and case series are needed to explore the prognosis of patients treated with DEB-TACE.

## Data availability statement

The original contributions presented in the study are included in the article/supplementary material. Further inquiries can be directed to the corresponding author.

## Ethics statement

All procedures performed in studies involving human participants were in accordance with the ethical standards of the institutional and/or national research committee and with the 1964 Helsinki declaration and its later amendments or comparable ethical standards. Written informed consent was obtained from the participant/patient(s) for the publication of this case report.

## Author contributions

ZQ: Writing – review & editing, Writing – original draft, Resources. JJ: Writing – original draft, Methodology, Data curation. SZ: Writing – review & editing, Conceptualization. SW: Writing – review & editing.
